# Severe Sepsis Secondary to Toxic Megacolon Revealing an Inflammatory Bowel Disease

**DOI:** 10.7759/cureus.51459

**Published:** 2024-01-01

**Authors:** Omar Mhammedi Alaoui, Badie Douqchi, Islam Bella, Imane Ghazi, Ilias Benaini, Ilias El Kadiri Boutchich, Ilyass Laaribi, Ghizlane El Aidouni, Houssam Bkiyar, Mohammed Bouziane, Brahim Housni

**Affiliations:** 1 Intensive Care Unit, Faculty of Medicine and Pharmacy of Oujda, Mohammed First University, Oujda, MAR; 2 Intensive Care Unit, Mohammed VI University Hospital, Oujda, MAR; 3 Intensive Care Unit, Mohammed First University, Oujda, MAR; 4 Anesthesiology - Critical Care Unit, Mohammed VI University Hospital, Oujda, MAR; 5 General Surgery A, Chu Mohammed 6, Oujda, MAR; 6 Intensive Care and Anesthesiology, Mohammed VI University Hospital, Oujda, MAR

**Keywords:** intensive care unit, inflammatory bowel disease, septic shock, sepsis, toxic megacolon

## Abstract

Patients with inflammatory bowel disease can present with numerous infectious complications, including intra-abdominal abscess, perforations of the intestine, fistula formation, and the occurrence of septicemia. Toxic megacolon (TM) is a potentially fatal complication of inflammatory bowel disease (IBD) and is associated with high morbidity and mortality. In this case report, we report a 49-year-old male patient who was admitted to the intensive care unit for the management of severe sepsis that was secondary to an inaugural toxic megacolon complicating a silent inflammatory bowel disease, with a Lichtiger score of 11. Nonresponse to anti-bacterial therapy, noradrenaline, and intravenous corticosteroid therapy required an emergency total colectomy. After surgery, the patient died because of his unresolved septic shock. Correct management of this condition requires an accurate assessment of the patient’s history, a correct physical examination, abdominal radiographs, and sigmoid coloscopy, and frequently requires surgery. The indications for surgery in cases of toxic megacolon, massive hemorrhage, perforation, peritonitis, or non-response to medical therapy are the most important ones. Patients with a history of inflammatory bowel disease are particularly prone to infectious complications since therapy for these inflammatory diseases is based on the use of immunosuppressive drugs and frequent abdominal surgeries.

## Introduction

Infections are well-documented complications of inflammatory bowel diseases [1]. These infections can manifest as local phenomena such as the formation of fistulae (perianal and enterovesical fistulae), especially in cases of Crohn’s disease [1], intra-abdominal abscesses, phlegmons caused by microperforation of the intestine, and sepsis [2,3]. The risk of infectious complications increases when the patient has a known history of inflammatory bowel disease, immunosuppressive drug intake, and/or a prior history of abdominal surgery [4,5]. Although rare, toxic megacolon is a fatal complication of inflammatory bowel disease. This condition is characterized by an acute colonic dilatation, with loss of haustration on radiologic examination [6,7]. In this work, we report a 49-year-old male patient who was admitted to the intensive care unit for the management of severe sepsis that was secondary to an inaugural toxic megacolon complicating a silent inflammatory bowel disease, with a Lichtiger score of 11. Nonresponse to anti-bacterial therapy, noradrenaline, and intravenous corticosteroid therapy required an emergency total colectomy.

## Case presentation

A 49-year-old male patient was admitted to the hospital after having acute-onset bloody diarrhea (8 bloody stools per day), with fever, rapid body weight loss (3 kg) over a period of two weeks, and diffuse severe abdominal pain. The patient reports no prior similar episodes, and he was not known to have inflammatory bowel disease. At admission to the emergency department, the patient was conscious with no neurological symptoms. He had a fever of 39 ℃ and low systolic blood pressure of 80 mmHg and the admission electrolyte exploration showed profound hypokalemia at 2.8 mmol/L. At the intensive care unit, a physical examination revealed a blood pressure of 80/40 mmHg, with a mean arterial pressure (MAP) of 53 mmHg, a heart rate of 121beats/min, a respiratory rate of 28 breaths/min, and oxygen saturation of 98%. Physical examination of the skin and the anal canal has revealed no specific findings. Abdominal examination revealed a diffuse sensibility with no tenderness.

Results of laboratory explorations are resumed in Table 1.

**Table 1:** Results of laboratory explorations in our patient

Parameter	Value in our patient	Normal range for our laboratory
Lactate level	40 mg/dL	4,5 – 19.8 mg/dL
Leucocyte count	7,500/μL	4500 – 11000/μL
Hemoglobin level	9.1 g/dL	13.8 – 17.2 g/dL
Platelet count	214000 /μL	150000 – 400000 /μL
Prothrombin	30% of the normal	85 – 100%
D-dimers level	20.8 μg/mL	<0.5 μg/mL
Creatinine blood level	1.89 mg/dL	0.7 – 1.3 mg/dL
Serum amylase	176 U/L	40 – 140 U/L
Lipase level	524 U/L	0 – 160 U/L
C-reactive protein	61.7 mg/L	<6 mg/L
Procalcitonin	100 ng/dL	< 1 ng/dL
Urinary leukocytes	20 white blood cells per high-power field	2 – 5 leukocytes/high-power field
Urine culture	No organisms found	

**Figure 1:**
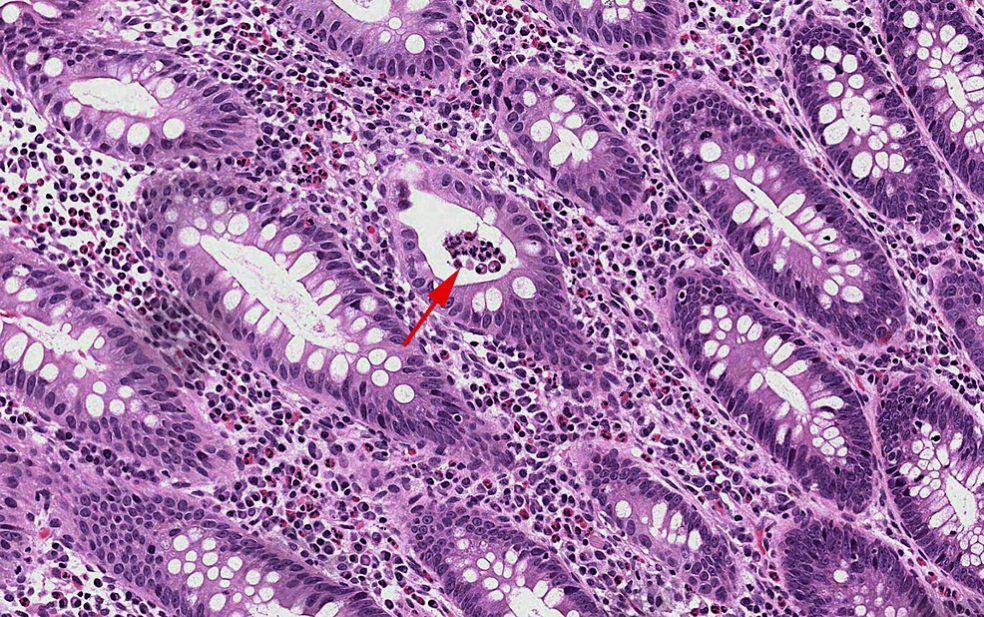
Microphotograph revealing a rectal mucosa with dense inflammatory infiltrate made of neutrophils and eosinophils Numerous cryptic abscesses were observed (Arrow). No cytomegalovirus inclusions were observed. (HE; 100X)

Thorax radiograph revealed a right lung atelectasis with no detection of signs of pneumonia. Flexible sigmoidoscopy was performed, revealing a hemorrhagic rectal and sigmoidal mucosa. A rectal biopsy was performed. Pathological assessment has revealed signs of inflammatory bowel disease with severe activity. Cytomegalovirus nuclear inclusions and amoeba bodies were absent (Figure 1).

**Figure 2:**
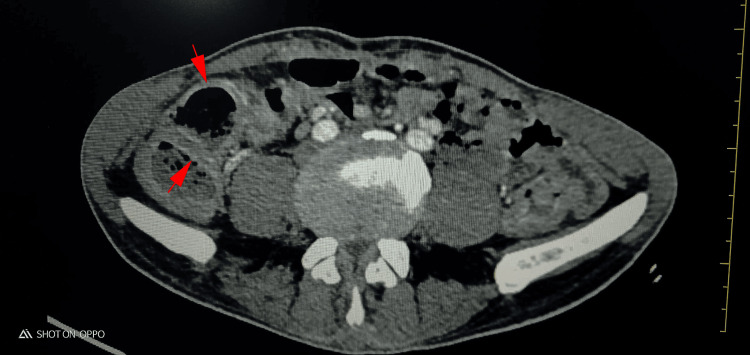
Abdominal computed tomography (CT) scan revealing diffuse parietal swelling (red arrow) and contrast intake in the mucosal layer No obvious abscesses or intestinal perforations were found.

An abdominal computed tomography (CT scan) scan revealed important and diffuse swelling of the whole colonic wall. The colonic dilatation exceeded 7 cm, with contrast intake in the mucosal layer. No obvious abscesses or intestinal perforations were found (Figure 2).

The diagnosis of septic shock secondary to acute severe colitis was established with a Lichtiger score of 11. The patient received anti-bacterial therapy based on ceftriaxone (2 g intravenous per day), metronidazole (50 mg/kg per day), and gentamicin (7 mg/kg, intravenous per day), noradrenaline (0.15 micrograms/kg/min), along with hypokalaemia correction. The patient also received intravenous corticosteroid therapy based on methylprednisolone at 0.8 mg/kg daily.

The patient presented no amelioration after 3 days of corticosteroid therapy, requiring, therefore, an emergency colectomy. Intraoperatively, diffuse thickening and dilation of the whole colon wall were identified with no skip colonic regions. After surgery, the patient died because of his unresolved septic shock.

## Discussion

Toxic megacolon is a fatal condition, with a prevalence that is generally underreported and that is known to increase with age. All ages and both genders can be affected by toxic megacolon [8].

In patients with known inflammatory bowel disease, half of the patients develop toxic megacolon during the first three months after the initial diagnosis of inflammatory bowel disease [9]. The most important causes of TM are ulcerative colitis (UC) and Crohn’s disease (CD) [10]. Other conditions known to potentially cause TM, include infectious etiologies such as Clostridium difficile colitis and cytomegalovirus colitis [11].

The mechanisms implicated in TM remain unclear, although evidence of defective smooth muscle contraction and lowered basal pressure of the colonic lumen are well-known mechanisms that are involved in TM pathogenesis [12].

On the clinical level, severe bloody diarrhea, as reported by our patient, is the most appealing symptom. Other symptoms include hypotension, tachycardia, fever, and abdominal distention [13]. Laboratory findings are often a reflection of systemic toxicity and include leukocytosis, metabolic alkalosis, and electrolyte level alteration [8].

In our case, signs of septic shock were present at the clinical and biological levels without the identification of any infectious focus. This was, for us, highly suggestive of the dissemination of bacteria to the bloodstream via the intestinal tract. This would be theoretically favored by an injured intestinal mucosa during episodes of toxic megacolon.

This hypothesis can be further supported by a possible occurrence of infection in the absence of classical infectious sources encountered in inflammatory bowel disease, namely, fistulas, abscesses, and phlegmons [2,3]. None of these elements could be identified in our patient. 

Current literature includes a case of an 11-year-old boy with a history of Crohn’s disease [1]. Our report indicates that the investigation of inflammatory bowel disease presence might be warranted by considering infectious complications and sepsis resulting from toxic megacolon. This holds true even when there is no history of immunosuppressive medication or surgical interventions.

Even in the absence of immunosuppressive drugs or a history of surgery, infection and secondary sepsis, as in our patient, can occur [1,4,5].

We believe that our reported case shows the fact that the occurrence of infectious complications and sepsis is possible even in the absence of immunosuppressive therapy. A suspicion of inflammatory bowel disease was possible through symptoms reported by the patient and through pathological assessment that could identify signs of this affection and the absence of intestinal pathologies such as amebiasis and cytomegalovirus.

## Conclusions

The risk of developing infectious complications is higher in patients with a documented history of inflammatory bowel disease, the use of immunosuppressive medications, or a previous abdominal surgery. While uncommon, toxic megacolon represents a potentially lethal complication of inflammatory bowel disease. This condition is marked by an abrupt enlargement of the colon, as evidenced by the absence of haustration on radiographic evaluation. Our study highlights the possibility that infectious complications and sepsis arising from toxic megacolon could serve as indicators for investigating the presence of inflammatory bowel disease, regardless of the absence of immunosuppressive medication or surgical history.
